# SFRP1 Negatively Modulates Pyroptosis of Fibroblast‐Like Synoviocytes in Rheumatoid Arthritis: A Review

**DOI:** 10.3389/fimmu.2022.903475

**Published:** 2022-06-20

**Authors:** Ping Jiang, Kai Wei, Cen Chang, Jianan Zhao, Runrun Zhang, Lingxia Xu, Yehua Jin, Linshuai Xu, Yiming Shi, Shicheng Guo, Steven J. Schrodi, Dongyi He

**Affiliations:** ^1^ Guanghua Clinical Medical College, Shanghai University of Traditional Chinese Medicine, Shanghai, China; ^2^ Department of Rheumatology, Shanghai Guanghua Hospital, Shanghai University of Traditional Chinese Medicine, Shanghai, China; ^3^ Department of Medical Genetics, School of Medicine and Public Health, University of Wisconsin-Madison, Madison, WI, United States; ^4^ Computation and Informatics in Biology and Medicine, University of Wisconsin-Madison, Madison, WI, United States; ^5^ Institute of Arthritis Research in Integrative Medicine, Academy of Traditional Chinese Medicine, Shanghai, China; ^6^ Department of Rheumatology, The Second Affiliated Hospital of Shandong University of Traditional Chinese Medicine, Jinan, China

**Keywords:** Secreted frizzled-related protein 1, rheumatoid arthritis, Wnt/β-catenin signaling pathway, Notch signaling pathway, pyroptosis, epigenetic

## Abstract

Secreted frizzled-related protein 1 (SFRP1) is a member of secretory glycoprotein SFRP family. As a primitive gene regulating cell growth, development and transformation, SFRP1 is widely expressed in human cells, including various cancer cells and fibroblast-like synoviocytes (FLS) of rheumatoid arthritis (RA). Deletion or silencing of SFRP1 involves epigenetic and other mechanisms, and participates in biological behaviors such as cell proliferation, migration and cell pyroptosis, which leads to disease progression and poor prognosis. In this review, we discuss the role of SFRP1 in the pathogenesis of RA-FLS and summarize different experimental platforms and recent research results. These are helpful for understanding the biological characteristics of SFRP1 in RA, especially the mechanism by which SFRP1 regulates RA-FLS pyroptosis through Wnt/β-catenin and Notch signaling pathways. In addition, the epigenetic regulation of SFRP1 in RA-FLS is emphasized, which may be considered as a promising biomarker and therapeutic target of RA.

## Introduction

Rheumatoid arthritis (RA) is an autoimmune-mediated chronic progressive disease and the synovium is the main target tissue. The main pathological features are persistent synovial hyperplasia and inflammation, which eventually lead to joint injury, cartilage destruction, and bone erosion (1, 2). Fibroblast-like synoviocytes (FLS) is a special group of cells in the synovial tissue. During the pathogenesis of RA, FLS show high proliferation, high invasiveness, and tumor-like changes, which play an important role in the development of RA (3, 4). Previous studies have shown that apoptosis and autophagy in FLS are closely related to the development of RA (5, 6). Unlike apoptosis, pyroptosis is a newly discovered form of programmed cell death that releases powerful immune cytokines such as IL-1β and IL-18 (7). In RA pathogenesis, the activation of inflammatory bodies, protease processing, and the release of inflammatory factors are related to abnormal synovium proliferation and bone destruction (8). Research on FLS pyroptosis may provide a new understanding of the pathogenesis of RA. The regulation of pyroptosis process can become a new treatment strategy for RA.

The Notch and Wnt/β-catenin signaling pathways are two highly conserved and functionally closely related pathways that coordinate and regulate cell growth, differentiation, and proliferation in various tissues and are involved in the development of various diseases (9). Secreted frizzled-related protein 1 (SFRP1) is a soluble protein that is highly restricted in tissue distribution. Part of its structure is highly homologous to the Frizzled (FZD) receptor of the Wnt/β-catenin signaling pathway; hence, it has the ability to bind to the Wnt protein and FZD receptor (10). Therefore, SFRP1 is considered a Wnt signaling pathway antagonist, which in turn interfere with Wnt signaling transduction and plays an important role in determining cell fate by regulating cell proliferation, differentiation, apoptosis, and pyroptosis (11). This regulation has also been studied in RA-FLS (12). In addition, some studies have found that SFRP1 can bind to recombinant A disintegrin and metalloproteinase 10 (ADAM10) protein and downregulate its activity in the Notch signaling transduction pathway, thus blocking the activation of Notch signaling (13).

One gene may act on different signaling pathways, and the regulatory role of SFRP1 in Wnt/β-catenin and Notch signaling pathways may be related to the pathological mechanisms of different diseases and may be associated with the process of pyroptosis. Functional connections among genes, signaling pathways, and pyroptosis may also exist in RA-FLS, and the crosstalk between different pathways may play an important role in the pathology and development of RA. In this review, we have added a new section on the pathological mechanisms of RA. This manuscript focuses on the relationship between SFRP1, Wnt/β-catenin signaling pathway, Notch signaling pathway, and pyroptosis, and their involvement in the pathogenesis of RA-FLS. The epigenetic regulation of SFRP1 could be a promising RA biomarker and a therapeutic target.

## Wnt/β-Catenin Signaling Pathway Regulates RA-FLS

**Figure 1:**
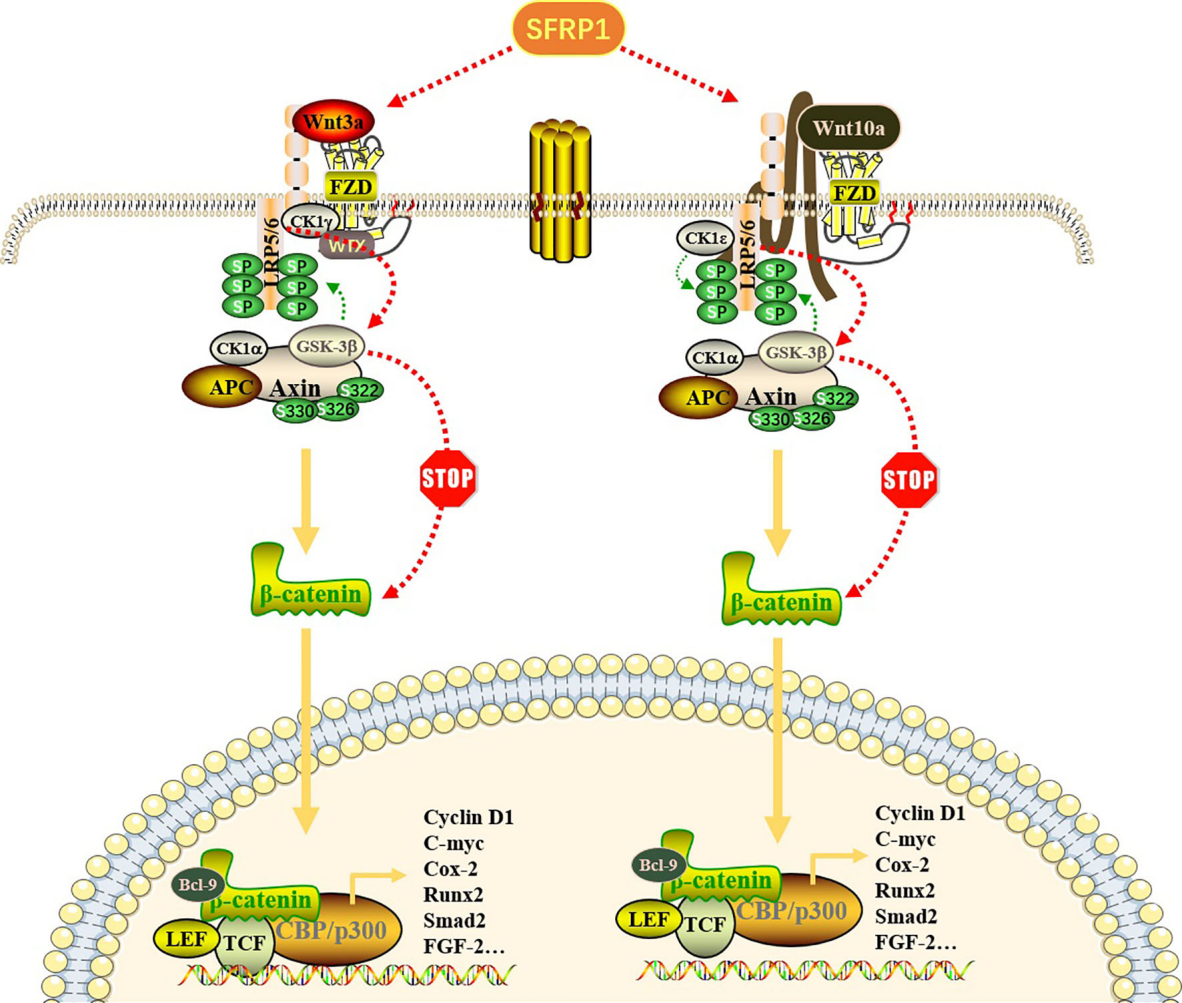
Effect of SFRP1 on Wnt/β-catenin signaling pathway. Wnt proteins (Wnt3a and Wnt10a) bind to FZD proteins located on the cell membrane to form a Wnt-FZD complex, which further binds to low-density lipoprotein receptor-related protein (LRP) 5/6 co-receptors, resulting in its cytoplasmic tail phosphorylation. When the pathway is not activated, β-catenin binds to the “destruction complex” composed of APC, GSK-3β, CK1α and Axin to promote its ubiquitin degradation. Once this pathway is activated, Axin dissociates from the destruction complex and binds to the phosphorylation site in the cytoplasmic tail of LRP. With the repositioning of Axin on LRP, the β-catenin released by the destruction complex is transported to the nucleus in the form of phosphorylation and binds to transcription factors, especially TCF and LEF, thereby regulating gene transcription and expression of related target genes. SFRP1 binds Wnt ligands through its CRD, thus preventing it from binding to FZD receptors, eliminating the accumulation of β-catenin and blocking the expression of downstream genes.

The Wnt signaling pathway is a complex signal transduction network that plays an important role in maintaining a balance between human growth and development (14). The Wnt/β-catenin signaling pathway belongs to the classical Wnt signaling pathway and is one of the most important and well-studied signaling pathways (15). The main components involved in this pathway include β-catenin, axon protein (Axin), transmembrane receptors (LRP5/6 and FZD), and T cell factor/lymphoid enhancer factor (TCF/LEF) (16) (**Figure 1** and **Table 1**). The classical Wnt signaling pathway mainly regulates the stability and accumulation of β-catenin in cells. The important effect of the Wnt/β-catenin signaling pathway on RA is reflected in the regulation of FLS activation and bone metabolism (20, 21). In RA patients, the FLS division rate is faster than that in normal people, and hyperproliferative FLS is a key indicator in joint synovitis. Activated FLS can produce pro-inflammatory factors and matrix metalloproteinases, cause inflammatory cell infiltration and pannus formation, and lead to the persistent destruction of cartilage and bone (22, 23). Recent studies have shown that the an increased expression of proteins such as Wnt3a, Wnt5a, and Wnt10a in RA-FLS (**Table 1**), activates the Wnt signaling pathway and downstream genes, and increases the expression of fibronectin, thereby promoting cell proliferation, migration, and survival, and promotes RA synovial tissue proliferation in the absence of pro-inflammatory factors (24, 25). In addition, the Wnt/β-catenin signaling pathway regulates immune system homeostasis. In normal circumstances, β-catenin can improve the survival rate of regulatory T cells (Tregs), while activating the Wnt canonical pathway under inflammatory conditions may inhibit Treg function, leading to an autoimmune response (26). In conclusion, regulation of the Wnt/β-catenin signaling pathway in the pathogenesis of RA is multi-level and multi-faceted.

**Table 1:** Differentially expressed genes involved in Wnt/β-catenin, Notch signaling pathway and pyroptosis in RA-FLS *(P<0.05)*.

Name	Gene	Beta	Reference
Wnt/β-catenin signling pathway	SFRP1	↓	GSE55457 (17) GSE55584 (17) GSE55235 (17) GSE89408 (18, 19)
	FZD1	↑	
	FZD2	↑	
	FZD6	↑	
	WNT5A	↑	
	TCF7	↑	
	LEF1	↑	
	MYC	↓	
	MAPK8	↓	
	BCL9	↑	
Notch signalling pathway	NOTCH1	↑	
	RBPJ	↑	
	HDAC1	↑	
	ADAM10	↑	
Pyroptosis	NLRP3	↑	
	CASP1	↑	
	CASP4	↑	
	CASP5	↑	
	GSDMD	↑	
	IL18	↑	

Beta↑ means high expression in RA-FLS, if beta↓, the gene is down-regulated in RA-FLS.

## Notch Signaling Pathway Regulates RA-FLS

**Figure 2:**
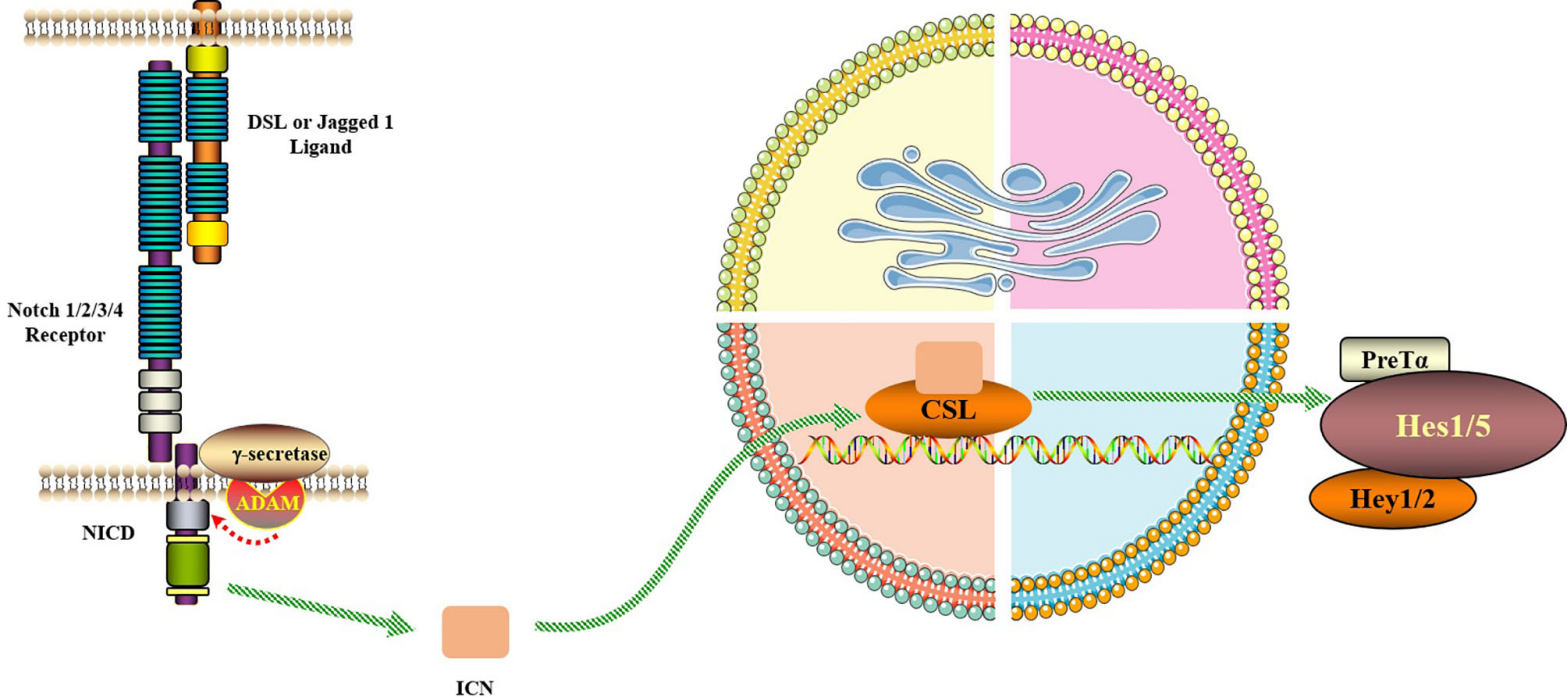
Notch signaling pathway. The Notch signaling pathway consists of Notch, Notch ligand (DSL or Jagged1/2) and CSL (a class of DNA-binding proteins, include RBPJ). Notch signaling is generated by the interaction of Notch ligands of neighboring cells with the receptor, initiating ADAM10-mediated proteolysis of the extracellular domain. After three times of cleavage, Notch protein is released into the cytoplasm from the NICD and enters the nucleus to bind to the transcription factor CSL to form the NICD/CSL transcriptional activation complex, which activates the target genes of the transcriptional inhibitory factor family such as Hes and Hey, and plays a biological role.

The Notch signaling pathway is a conserved and important signal transduction pathway that affects cell fate. It is widely expressed in many species including vertebrates and invertebrates. It is highly evolutionarily conserved and influences the proliferation and differentiation of almost all cell types (27, 28). The classical Notch signaling pathway is mainly composed of Notch, Notch ligand (DSL protein or Jagged1), and CSL (DNA-binding protein). Through protease hydrolysis, Notch protein fragments (NICD or ICN) with transcriptional regulatory activity are released and then bind to the transcription factor CSL to regulate downstream gene expression (29) (**Figure 2**). The atypical Notch signaling pathway induces the expression of different genes through crosstalk with signaling pathways, such as NF-κB, Wnt, and TGF-β (30, 31). Neighboring cells can transmit signals through the binding of Notch receptors to ligands, thereby expanding and stabilizing molecular differences between cells, ultimately determining cell fate and affecting tissue and organ formation (32). Notch signaling is an important pathway for communication between adjacent cells, regulates cell development (33, 34), and plays an important role in the pathogenesis of RA. Previous studies have focused on Notch1 signaling pathway activation and downstream target gene regulation, which affect cell proliferation, migration, and other processes in RA-FLS (**Table 1**), and interfere with Notch1 *via* siRNA to exert therapeutic effects (35–37). A recent single-cell RNA-sequencing study of synovial tissue found (38) that the expression of Notch3 and its downstream target genes was significantly upregulated in RA-FLS (**Table 1**). Notch3 signaling can drive both transcriptional and spatial gradients in FLS, contributing to the differentiation of FLS subtypes, and blocking this pathway helps attenuate arthritis development. In a mouse model, deletion of Notch3 or blockade of Notch3 signaling has also been shown to prevent joint damage in inflammatory arthritis. It is noteworthy that the ADAM10 protein, which is an important regulator of the Notch pathway, is involved in a variety of biological functions, including inflammation, apoptosis, cancer development, and autoimmunity (39). In the process of Notch signal transduction, binding of Notch with Notch ligands initiates proteolysis of the extracellular domain mediated by ADAM10, and induces the transcription of Notch target genes through a series of complex biological reactions, thus regulating the process of growth and development (40, 41). Comparing 292 osteoarthritis (OA) patients with healthy individuals, recent study has shown that the expression of ADAM10 in endothelial cells and FLS in RA biopsies is upregulated, suggesting that ADAM10 may be involved in the pathological development of RA (42). *In vitro* experiments showed that lowering the expression of ADAM10 by siRNA could inhibit the release of the proinflammatory cytokines TNF-α, IL-6, and IL-8 (43), improve the symptoms of arthritis, and reduce the level of vascular endothelial growth factor (VEGF) (42, 44). These findings suggest that inhibition of ADAM10 may effectively treat RA by inhibiting pro-inflammatory signal transduction and pannus formation in FLS. In addition to the importance of ADAM10 in Notch signaling, some substrates are related to ADAM10, such as Jagged1/2 (45). These results provide a molecular basis for targeted therapy of RA by regulating the Notch signaling pathway (46).

## Wnt/β-Catenin and Notch Signaling Pathway Crosstalk

Wnt/β-catenin and Notch signaling pathways have been independently shown to play a key role in regulating cell fate (47, 48). More evidence showed that there was a complex functional relationship between Wnt/β-catenin and Notch signaling (49). For example, GSK-3β kinase is responsible for the phosphorylation and inactivation of β-catenin in Wnt signaling pathway and can inhibit the transcription of Notch target genes by phosphorylating Notch2 (50, 51). Crosstalk between Wnt/β-catenin and Notch signaling can be observed in many different systems. In hematopoietic stem cell (HSC) formation, recent evidence suggests that Wnt signaling is helpful for Notch activity and formation by regulating the transcription of pre-embryonic Notch ligands, and the stability of undifferentiated HSC mediated by Wnt requires complete Notch signaling (52). In epidermal cells, β-catenin stimulates Notch signaling by inducing Jagged1 transcription, suggesting that the Notch signaling pathway plays a role downstream of the Wnt/β-catenin signaling pathway and determines the transformation of epidermal cells (53). In addition, many studies have elucidated the functional link between Wnt/β-catenin and Notch (53–57), however, this aspect has not been studied in details. At present, the interaction between the Wnt and Notch signaling pathways and its effect on the development of RA-FLS has not been documented. Whether there are biological mechanisms, such as the regulation of Notch2 by GSK-3β phosphorylation described earlier in RA-FLS, requires further research (13). SFRP1 can bind to ADAM10 metal protein in the Notch signaling pathway and downregulate its activity, thus blocking the activation of Notch signaling. Moreover, SFRP1 is also a suppressor gene of the Wnt/β-catenin pathway, which indicates that SFRP1 can inhibit both the Wnt/β-catenin and Notch signaling pathways. Can SFRP1 target RA through double inhibition? This was the direction of our team’s follow-up research.

## Pyroptosis Mechanism of RA-FLS

**Figure 3:**
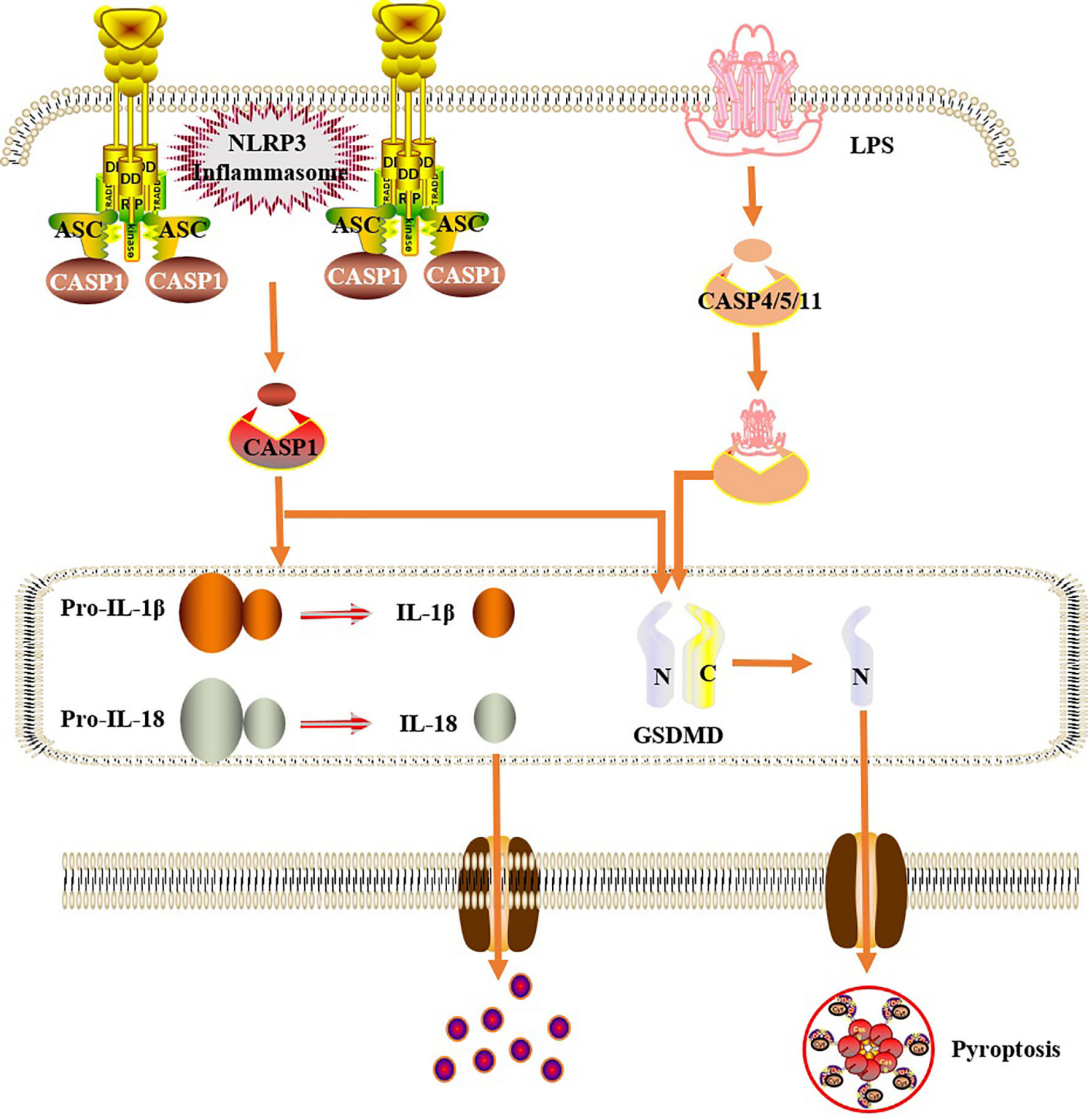
Cells pyroptosis mechanism. In the classical pathway, under the stimulation of bacteria, viruses and other signals, intracellular NLRs act as sensors to recognize these cognate ligand signals, and combine with the precursor of caspase1 through the adaptor protein ASC to form a multi-protein complex and activate caspase-1. Activated caspase-1 cleaves GSDMD to form a peptide segment containing the nitrogen-terminal active domain of GSDMD, which induces cell membrane perforation, cell rupture, release of contents, and inflammatory response. Moreover, activated caspase-1 also cleaves the precursors of IL-1β and IL-18 to form an active structure, which is released outside the cell, recruiting inflammatory cells to gather, expanding the inflammatory response and mediating cell pyroptosis. In the non-classical pathway, human caspase-4,5 and mouse caspase-11 can be directly activated by contact with bacterial LPS, then cleave GSDMD, and indirectly activate caspase-1, causing pyroptosis.

Pyroptosis, also known as the inflammatory necrosis of cells, is a type of programmed cell death. The process depends on the caspase, NOD-like receptor (NLR), and Gasdermins (GSDMs) protein families, accompanied by the release of a large number of pro-inflammatory factors, such as IL-1β and IL-18 (58). Pyroptosis is mainly characterized by the continuous expansion of cells until cell membrane ruptures, resulting in the release of cell contents and activation of a strong inflammatory response. The activation patterns include classical pathways mediated by activated inflammasomes, such as the NLRP3, and caspase-1, and non-classical pathways mediated by bacterial lipopolysaccharide (LPS) and caspase-4/5/11. In the classical pathway, activated inflammasomes can promote the self-cleavage of procaspase-1 into active caspase-1, which can cause the release of proinflammatory factors, such as IL-1β and IL-18, and cause GSDMD protein cleavage and pyroptosis. In the non-classical pathway, caspase-4/5/11 can directly recognize the oligomerization of bacterial LPS, thus causing pyroptosis by the cleavage of GSDMD (59–61) (**Figure 3** and **Table 1**). Studies have shown that excessive proliferation and pyroptosis of FLS play a key role in joint destruction and persistent inflammation in RA, and this pathological process is closely related to the participation of abnormal NLRP3 inflammasomes (NLRP3, ASC, and caspase-1 complex) (62). Increased levels of inflammatory cytokines such as IL-1β, TNF, IL-18, and IL6 in the serum and synovial fluid of patients with active RA were obtained and positively correlated with the level of NLRP3 (63, 64) (**Table 1**). In the CIA animal model, it was also found that the expression of the NLRP3 inflammasome was upregulated in synovial FLS, accompanied by a significant increase in MMP-1 levels in the supernatant. Upregulated NLRP3 can also promote the maturation and increase secretion of IL-1β and IL-18 through the cleavage of caspase-1 (65, 66) (**Figure 3**). In addition, IL-18 can induce FLS to secrete osteoclast cytokines, which play a role in bone resorption (67). These studies clearly show that inflammasomes and their downstream cytokines, IL-1β and IL-18, are involved in the pathogenesis of RA-FLS. Therefore, it is possible to block the activity of the NLRP3 inflammasome by blocking NLRP3, thus inhibiting pyroptosis of FLS, which will be discussed below.

## SFRP1 Regulates Dual Signaling Pathways to Mediate RA-FLS Pyroptosis

SFRP is a protein that can be folded into two independent domains, the N-terminal and C-terminal domains. The N-terminal cysteine-rich domain region (CRD) can bind to FZD receptors through disulfide bonds; therefore, SFRP1 can act as a regulator of the Wnt/β-catenin signaling pathway (68, 69). Given the critical role of the Wnt/β-catenin signaling pathway in the development of RA pathology, it is possible to block Wnt signaling pathway molecules to reduce the expression of inflammatory factors in RA synovial cells, including some secreted SFRP proteins which may inhibit inflammation by competitively binding to Wnt protein and down-regulating c-Jun N-terminal kinase (c-JNK) (70). In the SFRP family, SFRP1 has been widely studied in RA patients. SFRP1 negatively regulates the Wnt/β-catenin signal transduction pathway (71). SFRP1 interacts with Wnt protein or FZD receptor to eliminate the accumulation of β-catenin and block the expression of downstream genes by isolating Wnt, which is useful in inhibiting many biological processes, such as proliferation and apoptosis of RA-FLS (72, 73) (**Figure 1**). In addition, in the review by Claudel et al., researchers introduced in detail the role of sFRPs family members in cancer, bone and joint diseases, and summarized the different roles of each SFRP in pathophysiology, which have different effects on Wnt signaling pathway and different inflammation-related signals. Especially in the control of inflammatory response of RA, reducing the level of sFRP1 is a promising way to control the differentiation of Th17, especially when biological agents are ineffective. At the same time, it is also described that the expression of sFRPs in inflammatory synovium is regulated by epigenetics, and any decrease in sFRPs level may lead to self-persistence of joint inflammation (74). A similar inhibition was also observed in the Notch signaling pathway (13). In RA-FLS, SFRP1 can bind to the ADAM10 metal protein of the Notch signaling pathway and downregulate its activity, thus blocking the activation of Notch signaling. Of course, among the targets of Notch signaling pathway, the down-regulation of ADAM10 is not the only mechanism to interfere with Notch signaling pathway. It is possible that SFRP1 interacts with other targets to interfere with the Notch pathway, so the down-regulation of ADAM10 is one of the possibilities, and more findings need to be further studied. In addition, RNA-sequence was detected in synovial tissues of patients with RA and OA. The results of multiple studies showed that SFRP1 was expressed at low levels in RA (**Table 1**). Therefore, blocking downstream signaling pathways and genes by upregulating the expression of SFRP1 in RA-FLS would be helpful in the treatment of RA.

There is also a relationship between the Wnt/β-catenin and Notch signaling pathways and cell pyroptosis. Studies have shown that β-catenin interacts with NLRP3 and promotes its binding and that of ASC, thus activating the NLRP3 inflammasome and initiating the subsequent process of cell pyroptosis. When siRNA was used to inhibit β-catenin expression, activation of the NLRP3 inflammasome was also observed (75). This reveals a new role of β-catenin in the activation of the NLRP3 inflammasome and suggests that there is endogenous signaling crosstalk between the Wnt/β-catenin signaling pathway and NLRP3 inflammasome. Similarly, the relationship between the activation of Notch1 and NLRP3 was confirmed in the Notch signaling pathway. The expression levels of Notch1, NLRP3, and pro-inflammatory cytokines were detected in skin scar fibroblasts when compared to normal counterparts. The results showed that the expression levels of Notch1 and NLRP3 were higher in skin scar fibroblasts than in normal fibroblasts. Inhibition of Notch1 expression by siRNA transfection significantly inhibited the expression of NLRP3 inflammasome and related pro-inflammatory factors (76). These results confirm that Notch1 is a novel factor that activates the NLRP3 inflammasome. Inhibition of Notch1 can downregulate the activation of NLRP3, slow down chronic tissue injury and fibroblast differentiation in skin scars, and regulate the innate immune response.

**Figure 4:**
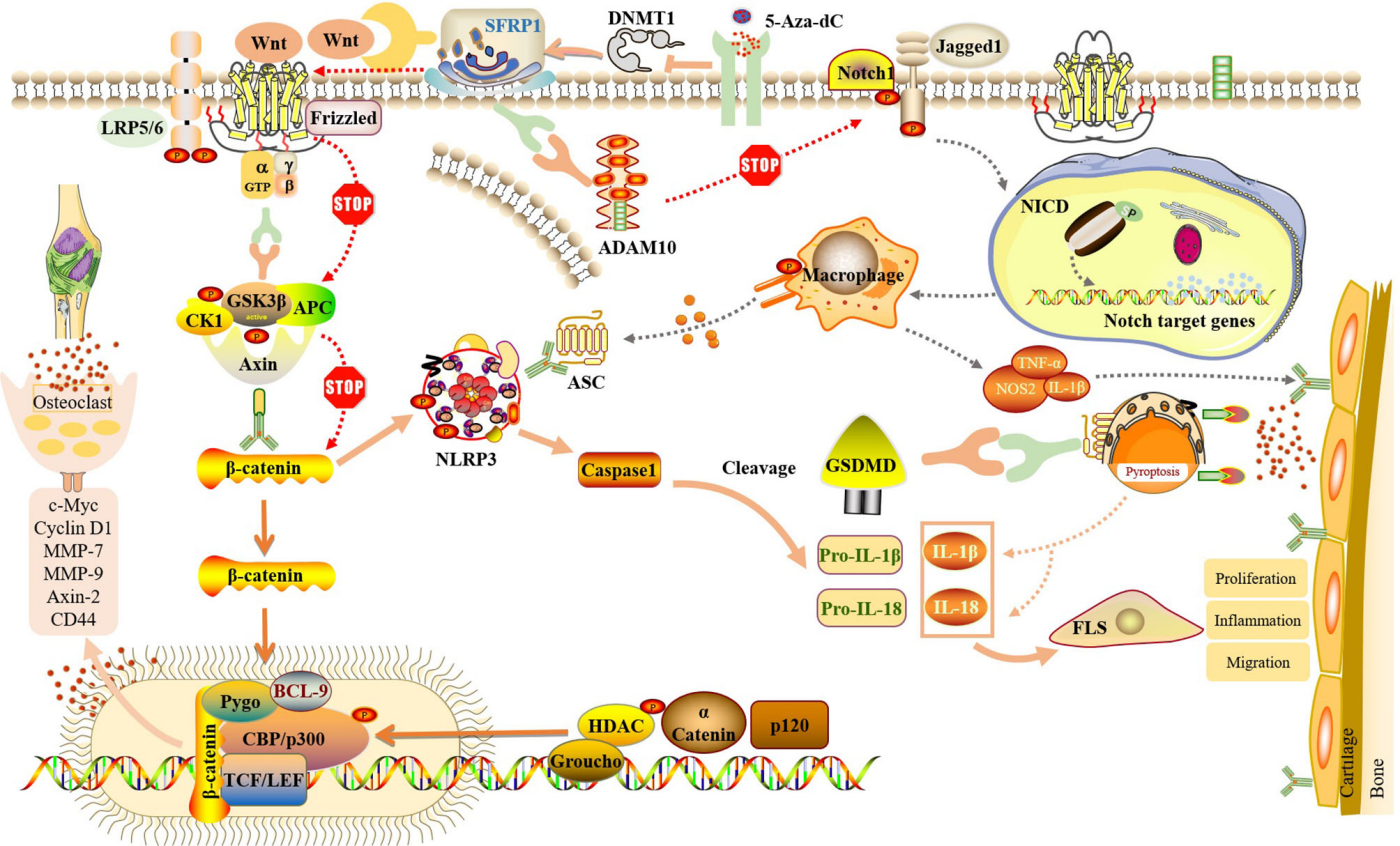
SFRP1 participate in NLRP3-mediated cell pyroptosis by regulating the dual signaling pathways of Wnt/β-catenin and Notch. By binding to Wnt protein and ADAM10 protein, SFRP1 negatively regulates Wnt/β-catenin and Notch signaling, blocks the activation of downstream proteins and the release of inflammatory factors, and reduces the inflammatory response of FLS and the destruction of articular cartilage. And indirectly inhibit the activation of NLRP3 inflammasome and block the occurrence of cell pyroptosis. Methylation inhibitor of 5-Aza-dC could inhibit the expression of DNMT, release SFRP1 hypermethylation and up-regulate the expression of SFRP1 in RA-FLS, thus negatively regulating Wnt/β-catenin and Notch signaling pathways.

In summary, although the regulation of NLRP3-mediated cell pyroptosis by the Wnt/β-catenin and Notch signaling pathways has not been carried out in RA-FLS, its key role is based on observation in other diseases. We propose the following hypothesis: in RA-FLS, SFRP1 participates in NLRP3-mediated cell pyroptosis by regulating the Wnt/β-catenin and Notch signaling pathways (**Figure 4**). Moreover, methylation detection in synovial tissue from knee joint in patients with RA and OA showed that several methylation sites of SFRP1 were hypermethylated in the synovial tissue from RA patients (77, 78). Therefore, the inhibition of hypermethylation of SFRP1 by methylation inhibitors is helpful in upregulating the expression of SFRP1, competitively inhibiting the signal transduction of Wnt/β-catenin and Notch, the release of downstream inflammatory cytokines, and NLRP3-mediated cell pyroptosis, thus playing a role in the treatment of RA.

## Discussion

FLS have always been considered an attractive therapeutic target for RA. However, no treatment that directly targets FLS has been approved. In this review, we systematically explain how SFRP1, Wnt/β-catenin signaling, Notch signaling, and cell pyroptosis independently affect the development of RA-FLS. Based on these theories, we propose that in RA-FLS, SFRP1 participates in NLRP3-mediated pyroptosis by regulating the Wnt/β-catenin and Notch signaling pathways, thereby affecting the progression of RA. Moreover, a preliminary study showed that SFRP1 was hypermethylated in RA synovial tissues. Thus, SFRP1 may serve as a potential target for RA treatment. Through the promotion of SFRP1, it is highly expressed in RA-FLS, so as to observe whether it can inhibit the activation of Wnt/β-catenin and Notch signaling pathway and the occurrence of pyroptosis, whether it can improve the inflammatory microenvironment of joint synovium and alleviate the symptoms of RA, which will be our main research work. In addition, to verify this hypothesis, our team is also planning to use traditional Chinese medicine, *Tripterygium wilfordii* Hook F, or methylation inhibitors and further develop drugs that potentially target SFRP1 to fill in the gaps related to RA-FLS therapy.

## Author Contributions

PJ and KW was responsible for the collection, collation of data, and writing of the original manuscript. CC, JZ, RZ, LXX, YJ, LSX, and YS were accountable for collection of data. SG, SS and DH were responsible for concept development and manuscript revision. All authors reviewed and accepted the final version of the manuscript. All authors contributed to the article and approved the submitted version.

## Funding

This work was funded by the National Natural Science Funds of China (82074234 and 82071756), Shanghai Chinese Medicine Development Office, National Administration of Traditional Chinese Medicine, Regional Chinese Medicine (Specialist) Diagnosis and Treatment Center Construction Project-Rheumatology, State Administration of Traditional Chinese Medicine, National TCM Evidence-Based Medicine Research and Construction Project, Basic TCM Evidence-Based Capacity Development Program, Shanghai Municipal Health Commission, and East China Region-based Chinese and Western Medicine Joint Disease Specialist Alliance.

## Conflict of Interest

The authors declare that the research was conducted in the absence of any commercial or financial relationships that could be construed as potential conflicts of interest.

## Publisher’s Note

All claims expressed in this article are solely those of the authors and do not necessarily represent those of their affiliated organizations, or those of the publisher, the editors and the reviewers. Any product that may be evaluated in this article, or claim that may be made by its manufacturer, is not guaranteed or endorsed by the publisher.

## Abbreviations

RA, rheumatoid arthritis; SFRP1, Secreted frizzled-related protein 1; FLS, fibroblast-like synoviocytes; FZD, Frizzled; ADAM10, recombinant A Disintegrin And Metalloprotease 10; Axin, axon protein; TCF, T cell factor; LEF, lymphoid enhancer factor; LRP, low density lipoprotein receptor related protein; Treg, regulatory T cells; DSL, Diselenide–Selenoester Ligation; CSL, Calpastatin domain L; JAG1, Jagged1; NICD, notch intracellular domain; NF-κB, nuclear factor kappa-B; TGF-β, transforming growth factor-β; TNF-α, tumor necrosis factor-α; IL, interleukin; VEGF, vascular endothelial growth factor; GSK-3β, Glycogen synthase kinase-3β; HSC, hematopoietic stem cell; LPS, lipopolysaccharide; NLRP3, NOD-like receptor thermal protein domain associated protein 3; ASC, apoptosis-associated speck-like protein containing a C-terminal caspase activation and recruitment domain; GSDM, Gasdermin; CIA, collagen inducedarthritis; MMP-1, matrix metalloproteinase 1; CRD, cysteine-rich domain; c-JNK, c-Jun N-terminal kinase; OA, Osteoarthritis
